# Outcomes of early versus delayed invasive strategy in older adults with non-ST-segment elevation myocardial infarction

**DOI:** 10.1038/s41598-022-15593-w

**Published:** 2022-07-06

**Authors:** Yong Hoon Kim, Ae-Young Her, Seung-Woon Rha, Cheol Ung Choi, Byoung Geol Choi, Ji Bak Kim, Soohyung Park, Dong Oh Kang, Ji Young Park, Sang-Ho Park, Myung Ho Jeong

**Affiliations:** 1 Division of Cardiology, Department of Internal Medicine, Kangwon National University School of Medicine, 156 Baengnyeong Road, Chuncheon, Gangwon 24289 Republic of Korea; 2 Cardiovascular Center, Korea University Guro Hospital, 148, Gurodong-ro, Guro-gu, Seoul, 08308 Republic of Korea; 3 Cardiovascular Research Institute, Korea University College of Medicine, Seoul, Republic of Korea; 4 Division of Cardiology, Department of Internal Medicine, Cardiovascular Center, Nowon Eulji Medical Center, Eulji University, Seoul, Republic of Korea; 5 Cardiology Department, Soonchunhyang University Cheonan Hospital, Cheonan, Republic of Korea; 6 Department of Cardiology, Cardiovascular Center, Chonnam National University Hospital, Gwangju, Republic of Korea

**Keywords:** Cardiology, Medical research

## Abstract

We evaluated the 3-year clinical outcomes following early invasive (EI) and delayed invasive (DI) strategies in older adults with non-ST-segment elevation myocardial infarction (NSTEMI) undergoing successful new-generation drug-eluting stents (DESs) implantation to reflect current real-world practice. Overall, 2437 older adults (age, ≥ 65 years) with NSTEMI were recruited from the Korea Acute Myocardial Infarction Registry-National Institute of Health. They were divided into two groups: EI (n = 1750) and DI (n = 687). The primary clinical outcome was the occurrence of major adverse cardiac and cerebrovascular events (MACCEs), defined by all-cause death, recurrent MI, any repeat coronary revascularization, and stroke. The secondary clinical outcome was stent thrombosis (ST). After multivariable-adjusted and propensity score-matched analyses, the primary and secondary clinical outcomes were not significantly different between the EI and DI groups. Even after the analysis was confined to those having complex lesions, these major clinical outcomes were similar between these two groups. The EI and DI strategies in older adults with NSTEMI receiving new-generation DES showed comparable results.

**Clinical Trial Registration:** URL: http://cris.nih.go.kr/cris/en/; Unique identifier: KCT0000863.

In patients with non-ST-segment elevation (STE) acute coronary syndrome (NSTE-ACS), an early invasive (EI) strategy is defined as coronary angiography (CAG) and percutaneous coronary intervention (PCI) performed within 24 h of hospital admission^1,2^. The European guideline recommends an EI strategy in patients with a high-risk (≥ 1) criterion^1^. The American College of Cardiology/American Heart Association guideline recommends an EI strategy for initially stabilized high-risk patients with NSTE-ACS and a delayed invasive (DI) strategy defined as a reasonable strategy for high/intermediate risk patients (class IIa and level of evidence B)^1,2^. The preference for EI strategy in patients with NSTE-myocardial infarction (NSTEMI) in the European and American guidelines are based on the result of the Timing of Intervention in Acute Coronary Syndrome (TIMACS) trial^3^. The data from a recent registry^4^ showed that in high-risk (Global Registry of Acute Coronary Events [GRACE] score ≥ 140) NSTE-ACS patients, early CAG was associated with significantly reduced mortality rate (HR 0.79; 95% CI 0.62–0.98). In another study, the EI strategy did not significantly reduce the risk of death or MI except for recurrent ischemia and the duration of in-hospital stay^5^. Hence, the optimal timing of PCI in NSTEMI has not been conclusively defined. For NSTE-ACS, age was an important determinant of outcomes in those patients^6,7^. However, the published data concerning the results of an EI strategy in the context of the older patients with NSTEMI are limited and are the subject of this study^1^. Tegn et al. reported that invasive strategy was superior to a conservative strategy for the reduction of MI, urgent revascularization, stroke, and death in patients aged ≥ 80 years with NSTE-ACS^8^. Unfortunately, the majority of the previous studies did not confine the study population to patients who received successful PCI or those who received new-generation drug-eluting stents (DESs)^3,6,7^. Currently, the new-generation DESs have nearly replaced bare-metal stents and first-generation DES for routine PCI; the new-generation DES is more effective than first-generation DES in reducing major clinical outcomes in patients with acute MI (AMI)^9^. Although we believe that these previous studies^3,6,7^ are valuable for estimating comparative clinical outcomes among different treatment strategies (EI, DI, or conservative treatment) in patients with NSTE-ACS, their findings have some limitations with respect to the current real-world practices. Hence, in this study, we evaluated the 3-year major clinical outcomes between the EI and DI strategies in older adults with NSTEMI undergoing successful new-generation DES implantation.

## Results

### Baseline characteristics

**Figure 1:**
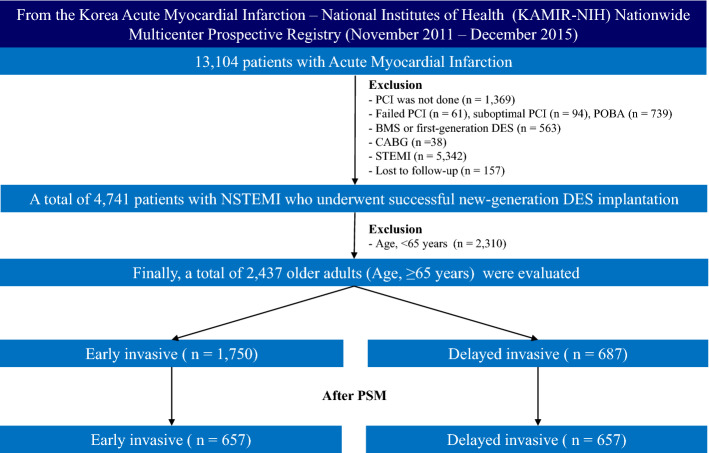
Flowchart. *PCI* percutaneous coronary intervention, *POBA* plain old balloon angioplasty, *BMS* bare-metal stent, *DES* drug-eluting stent, *CABG* coronary artery bypass graft, *STEMI* ST-segment elevation myocardial infarction, *NSTEMI* non-STEMI, PSM propensity score-matched analysis.

Figure 1 shows the flow chart of this study. Table 1 shows the baseline, laboratory, angiographic, and procedural characteristics of the study population. The mean values of left ventricular ejection fraction (LVEF), peak creatine kinase myocardial band (CK-MB), and peak troponin-I, and the number of current smokers, and the prescription rates of ticagrelor, angiotensin converting enzyme inhibitors (ACEIs) or angiotensin receptor blockers (ARBs) as discharge medications, multivessel disease and patients with pre-PCI thrombolysis in myocardial infarction (TIMI) flow grade 0/1 were higher in the EI group than in DI. In contrast, the patients who had Killip class ≥ 3, had reduced renal function (estimated glomerular filtration rate [eGFR], < 60 mL/min/1.73 m^2^), and received clopidogrel as discharge medication; mean value of serum creatinine and mean number of deployed stents; the use of intravascular ultrasound/optical coherent tomography/fractional flow rate were higher in the DI group than in EI (Table 1).

**Table 1:** Baseline clinical, laboratory, angiographic and procedural characteristics.

Variables	All patients (n = 2437)			SD	Propensity score-matched patients (n = 1314)			SD
	Early invasive (n = 1750)	Delayed invasive (n = 687)	p value		Early invasive (n = 657)	Delayed invasive (n = 657)	p value	
Male, n (%)	1017 (58.1)	397 (57.4)	0.731	0.14	359 (54.6)	379 (57.7)	0.291	− 0.62
Age, years	74.0 ± 5.9	74.7 ± 6.0	0.009	− 1.18	74.7 ± 6.3	74.6 ± 6.0	0.714	0.16
LVEF, %	52.6 ± 10.8	51.3 ± 12.3	0.019	1.12	51.8 ± 11.2	51.6 ± 12.1	0.744	0.17
BMI, kg/m^2^	23.2 ± 3.2	23.4 ± 3.3	0.055	− 0.62	23.4 ± 3.3	23.4 ± 3.2	0.780	0.15
SBP, mmHg	132.2 ± 25.7	137.9 ± 27.1	< 0.001	− 2.16	137.7 ± 27.4	137.2 ± 26.7	0.972	0.18
DBP, mmHg	78.5 ± 14.5	80.0 ± 15.2	0.029	− 1.01	79.9 ± 15.3	79.9 ± 15.1	0.578	− 0.02
Cardiogenic shock	88 (5.0)	31 (4.5)	0.676	0.23	23 (3.5)	30 (4.6)	0.400	− 0.56
Symptom-to-door time, h	8.0 (3.0–27.9)	8.7 (2.7–47.2)	0.031	− 1.03	8.0 (3.2–40.7)	8.1 (3.0–30.4)	0.578	− 0.31
Killip class ≥ 3	276 (15.8)	123 (17.9)	0.202	− 0.56	110 (16.7)	116 (17.7)	0.715	− 0.27
Hypertension, n (%)	1144 (65.4)	460 (67.0)	0.458	− 0.34	430 (65.4)	440 (67.0)	0.600	− 0.34
Diabetes mellitus, n (%)	636 (36.3)	249 (36.2)	0.964	0.02	241 (36.7)	236 (35.9)	0.819	0.17
Dyslipidemia, n (%)	164 (9.4)	89 (13.0)	0.012	− 1.14	74 (11.3)	80 (12.2)	0.668	− 0.28
Previous MI, n (%)	154 (8.8)	55 (8.0)	0.574	0.29	53 (8.1)	50 (7.6)	0.837	0.19
Previous PCI, n (%)	222 (12.7)	91 (13.2)	0.737	− 0.15	82 (12.5)	84 (12.8)	0.934	− 0.09
Previous CABG, n (%)	20 (1.1)	10 (1.5)	0.542	− 0.35	10 (1.5)	8 (1.2)	0.813	0.26
Previous HF, n (%)	38 (2.2)	18 (2.6)	0.548	− 0.26	16 (2.4)	17 (2.6)	0.860	− 0.13
Previous stroke, n (%)	143 (8.2)	66 (9.6)	0.261	− 0.49	62 (9.4)	57 (8.7)	0.701	0.24
Current smokers, n (%)	344 (19.7)	109 (15.9)	0.032	0.99	98 (14.9)	105 (16.0)	0.647	− 0.30
Peak CK-MB, mg/dL	21.6 (6.7–82.6)	13.7 (5.1–42.3)	< 0.001	2.25	15.3 (5.7–45.9)	17.6 (6.0–57.8)	0.887	0.09
Peak Troponin-I, ng/mL	10.9 (2.2–23.0)	4.7 (1.1–19.5)	< 0.001	2.18	5.8 (1.4–21.9)	6.1 (1.7–22.9)	0.830	0.11
Blood glucose, mg/dL	162.1 ± 76.6	164.3 ± 82.9	0.529	− 0.28	162.8 ± 76.4	163.1 ± 82.8	0.944	− 0.04
Hs-CRP (mg/dL)	2.19 ± 7.21	2.40 ± 9.71	0.617	− 0.25	2.51 ± 9.9	2.39 ± 9.8	0.838	0.12
Serum creatinine (mg/L)	1.17 ± 1.18	1.30 ± 1.37	0.025	− 1.02	1.28 ± 1.57	1.29 ± 1.36	0.943	− 0.07
eGFR < 60 mL/min/1.73 m^2^, n (%)	654 (37.4)	302 (44.0)	0.003	− 1.35	282 (42.9)	280 (42.6)	0.911	0.06
Total cholesterol, mg/dL	170.1 ± 43.4	171.8 ± 44.5	0.392	− 0.39	172.1 ± 44.4	171.4 ± 44.2	0.786	0.16
Triglyceride, mg/L	109.1 ± 75.3	112.0 ± 81.6	0.418	− 0.37	113.4 ± 94.1	112.0 ± 82.6	0.775	0.16
HDL cholesterol, mg/L	43.1 ± 11.6	44.5 ± 12.2	0.006	− 1.18	43.8 ± 11.8	44.3 ± 12.0	0.468	− 0.42
LDL cholesterol, mg/L	106.3 ± 34.7	105.4 ± 35.9	0.555	0.25	106.1 ± 35.9	105.7 ± 35.6	0.808	0.11
GRACE risk score	154.3 ± 36.9	156.0 ± 37.5	0.310	− 0.46	154.0 ± 36.7	155.8 ± 37.6	0.387	− 0.48
> 140, n (%)	1099 (62.8)	430 (62.6)	0.924	0.04	403 (61.3)	406 (61.8)	0.911	− 0.10
Atrial fibrillation, n (%)	104 (5.9)	47 (6.8)	0.402	− 0.37	38 (5.8)	45 (6.8)	0.496	− 0.41
ST-depression, n (%)	433 (24.7)	169 (24.6)	0.958	0.02	158 (24.0)	165 (25.1)	0.701	− 0.26
T-wave inversion, n (%)	400 (22.9)	163 (23.7)	0.669	− 0.19	162 (24.7)	155 (23.6)	0.699	0.26
**Discharge medications, n (%)**								
Aspirin, n (%)	1726 (98.6)	676 (98.4)	0.668	0.16	649 (98.8)	647 (98.5)	0.635	0.26
Clopidogrel, n (%)	1337 (76.4)	565 (82.2)	0.002	− 1.44	552 (84.0)	539 (82.0)	0.378	0.53
Ticagrelor, n (%)	308 (17.6)	87 (12.7)	0.003	1.37	78 (11.9)	87 (13.2)	0.505	− 0.39
Prasugrel, n (%)	81 (4.6)	24 (3.5)	0.267	0.56	19 (2.9)	21 (3.2)	0.873	− 0.17
BBs, n (%)	1421 (81.2)	569 (82.8)	0.383	− 0.41	547 (83.3)	545 (83.0)	0.941	0.08
ACEIs or ARBs, n (%)	1431 (81.8)	534 (77.7)	0.023	1.02	528 (80.4)	518 (78.8)	0.538	0.40
Statin, n (%)	1621 (92.6)	629 (91.6)	0.398	0.37	609 (92.7)	603 (91.8)	0.606	0.34
Anticoagulant, n (%)	53 (3.0)	25 (3.6)	0.444	− 0.35	20 (3.0)	25 (3.8)	0.544	− 0.44
**Infarct-related artery**								
Left main, n (%)	60 (3.4)	26 (3.8)	0.714	− 0.21	24 (3.7)	26 (4.0)	0.886	− 0.16
LAD, n (%)	744 (42.5)	305 (44.4)	0.399	− 0.38	297 (45.2)	291 (44.3)	0.781	0.18
LCx, n (%)	431 (24.6)	150 (21.8)	0.154	0.66	145 (22.1)	142 (21.6)	0.894	0.12
RCA, n (%)	515 (29.4)	206 (30.0)	0.786	− 0.13	191 (29.1)	198 (30.1)	0.717	− 0.22
Multivessel disease, n (%)	1052 (60.1)	456 (66.4)	0.005	− 1.33	436 (66.4)	434 (66.1)	0.953	0.06
ACC/AHA type B2/C lesions	1496 (85.5)	587 (85.4)	0.979	0.03	565 (86.0)	562 (85.5)	0.875	0.14
Pre-PCI TIMI flow grade 0/1	684 (39.1)	209 (30.4)	< 0.001	1.83	211 (32.1)	202 (30.7)	0.635	0.30
GP IIb/IIIa inhibitor, n (%)	148 (8.5)	47 (6.8)	0.213	0.64	49 (7.5)	46 (7.0)	0.831	0.19
Thrombus aspiration, n (%)	204 (11.7)	39 (5.7)	< 0.001	2.14	46 (7.0)	39 (5.9)	0.501	0.45
Transradial approach, n (%)	839 (47.9)	327 (47.6)	0.893	0.06	301 (45.8)	315 (47.9)	0.472	− 0.42
IVUS/OCT, n (%)	360 (20.6)	186 (27.1)	0.001	− 1.53	165 (25.1)	168 (25.6)	0.899	− 0.11
FFR, n (%)	27 (1.5)	23 (3.3)	0.007	− 1.18	18 (2.7)	19 (2.9)	0.868	− 0.12
IABP or ECMO, n (%)	40 (2.3)	9 (1.3)	0.149	0.75	11 (1.7)	9 (1.4)	0.822	0.24
**Drug-eluting stents**								
ZES, n (%)	399 (22.8)	163 (23.7)	0.631	− 0.21	154 (23.4)	156 (23.7)	0.948	− 0.07
EES, n (%)	948 (54.2)	360 (52.4)	0.443	0.36	344 (52.4)	348 (53.0)	0.868	− 0.12
BES, n (%)	348 (19.9)	152 (22.1)	0.220	− 0.54	151 (23.0)	143 (21.8)	0.643	0.29
Others, n (%)	55 (3.1)	12 (1.7)	0.072	0.92	8 (1.2)	10 (1.5)	0.813	− 0.26
Stent diameter (mm)	3.04 ± 0.40	3.03 ± 0.41	0.366	0.25	3.02 ± 0.40	3.03 ± 0.41	0.748	− 0.25
Stent length (mm)	30.1 ± 14.4	31.2 ± 15.1	0.100	− 0.75	31.8 ± 15.2	31.1 ± 14.9	0.447	0.47
Number of stents	1.21 ± 0.46	1.26 ± 0.50	0.042	− 1.04	1.26 ± 0.50	1.25 ± 0.49	0.868	0.20

Values are means ± standard deviation or median (interquartile range) or numbers and percentages. The p values for continuous data were obtained from the unpaired t-test. The p values for categorical data from chi-square or Fisher’s exact test. *LVEF* left ventricular ejection fraction, *BMI* body mass index, *SBP* systolic blood pressure, *DBP,* diastolic blood pressure, *MI* myocardial infarction, *PCI* percutaneous coronary intervention, *CABG* coronary artery bypass graft, *HF* heart failure, *CK-MB* creatine kinase myocardial band, *Hs-CRP* high sensitivity C-reactive protein, *eGFR* estimated glomerular filtration rate, *HDL* high-density lipoprotein, *LDL* low-density lipoprotein, *GRACE* Global Registry of Acute Coronary Events, *BBs* ß-blockers, *ACEIs* angiotensin-converting enzyme inhibitors, *ARBs* angiotensin receptor blockers, LAD left anterior descending artery, *LCx* left circumflex artery, *RCA* right coronary artery, *ACC/AHA* American College of Cardiology/American Heart Association, *TIMI* thrombolysis in myocardial infarction, *GP* glycoprotein, *IVUS* intravascular ultrasound, *OCT* optical coherence tomography, *FFR* fractional flow reserve, *IABP*, intra-aortic balloon pump, *ECMO*, extracorporeal membrane oxygenation, *ZES* zotarolimus-eluting stent, *EES* everolimus-eluting stent, *BES* biolimus-eluting stent.

### Clinical outcomes

**Figure 2:**
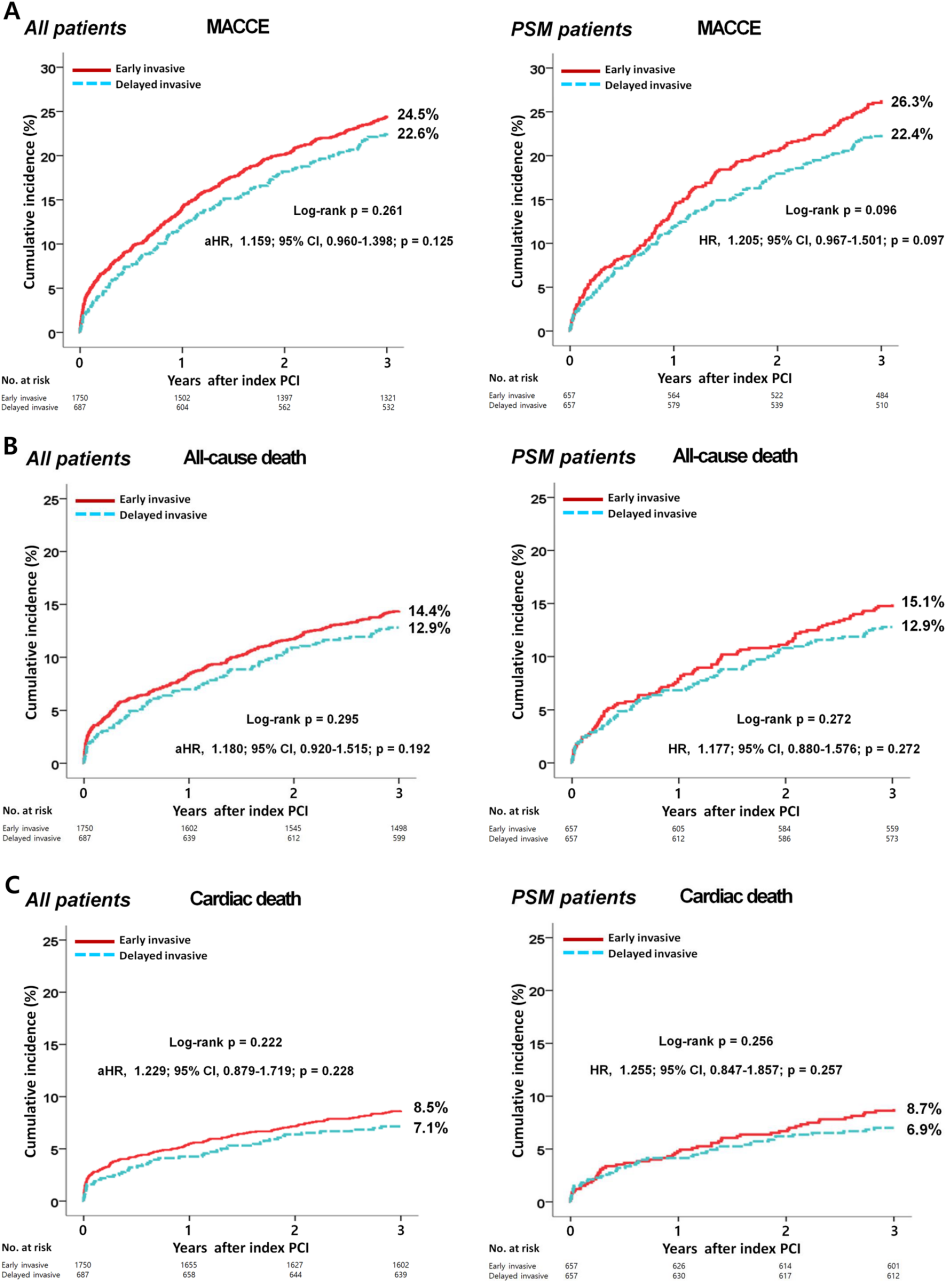

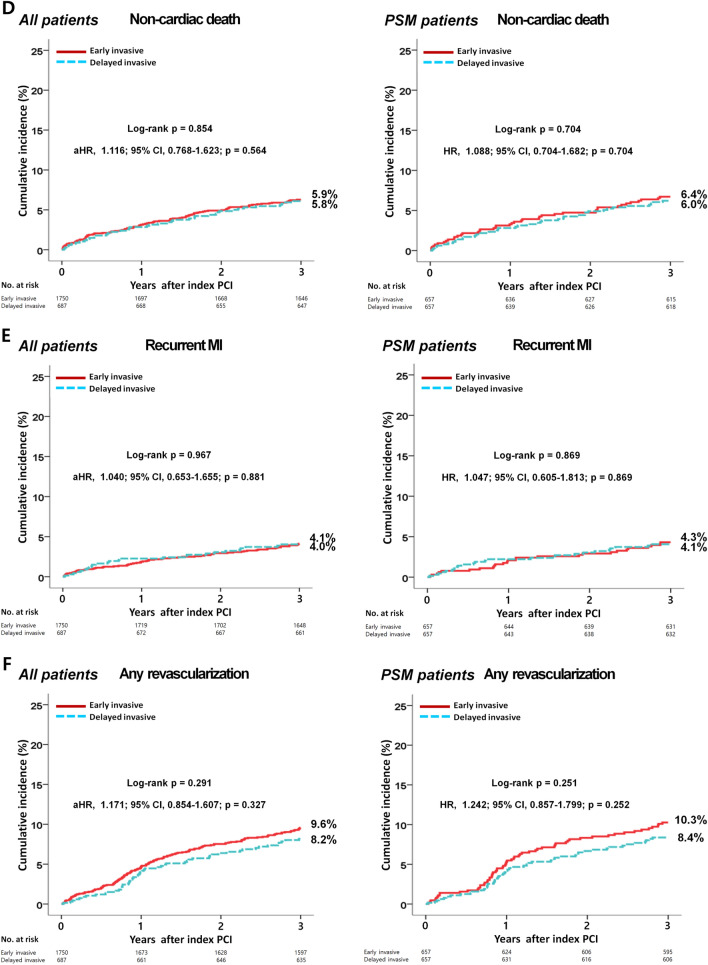

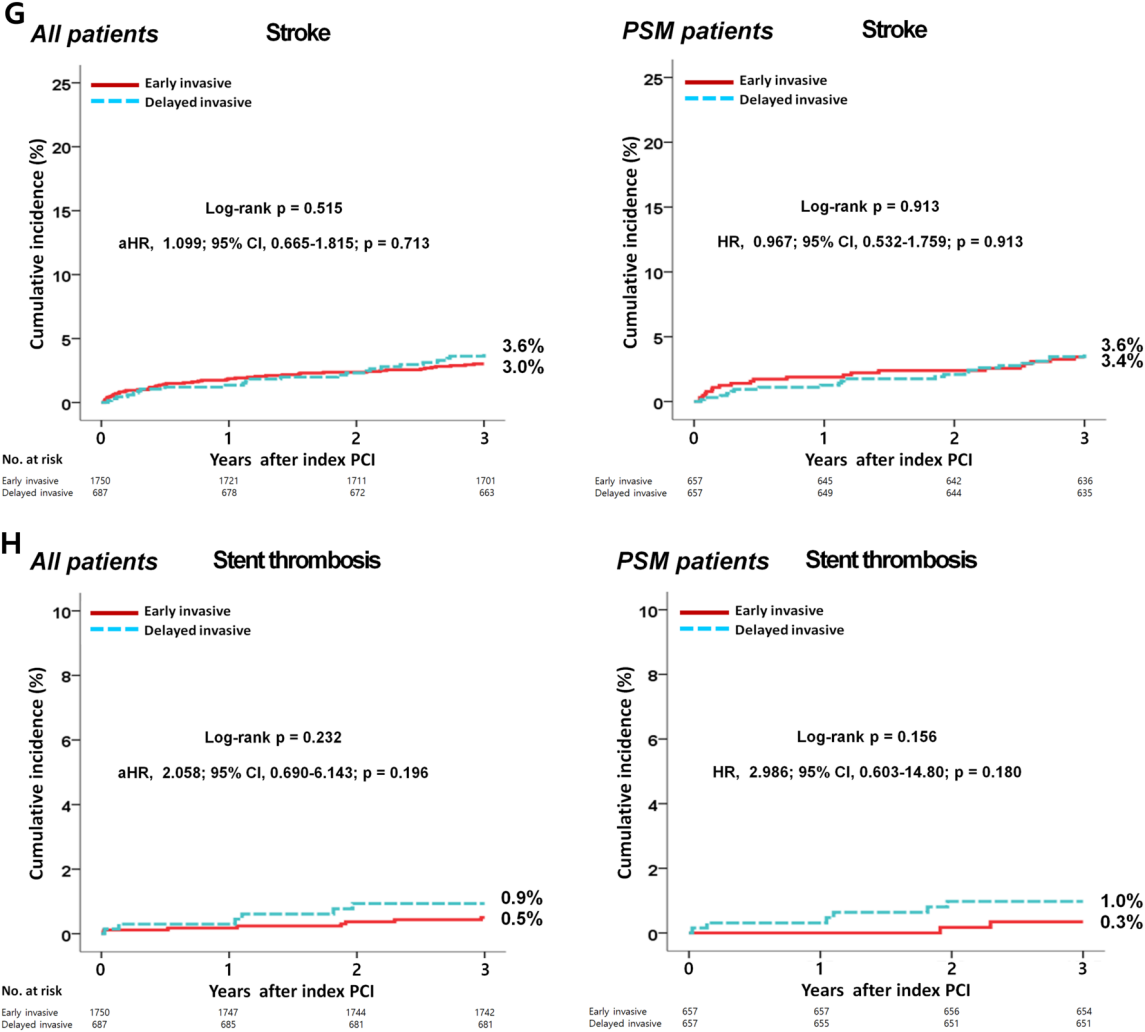
Kaplan–Meier curved analysis for MACCE (**A**), all-cause death (**B**), cardiac death (**C**), non-cardiac death (**D**), recurrent MI (**E**), any repeat revascularization (**F**), stroke (**G**), and stent thrombosis (**H**). *MACCE* major adverse cardiac and cerebrovascular events, *MI* myocardial infarction, *PSM* propensity score-matched, *HR* hazard ratio, *aHR* adjusted hazard ratio, *CI* confidence interval.

**Figure 3:**
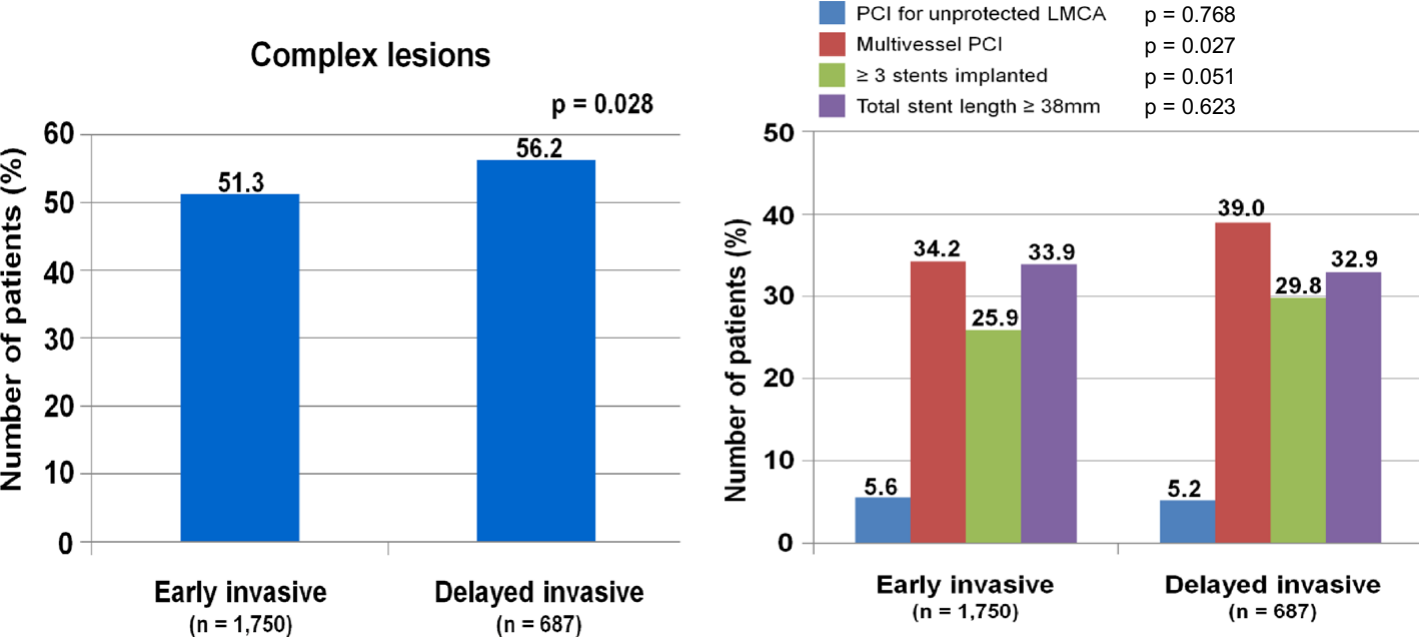
Distribution of complex lesions. *PCI* percutaneous coronary intervention, *LMCA* left main coronary artery.

**Figure 4:**
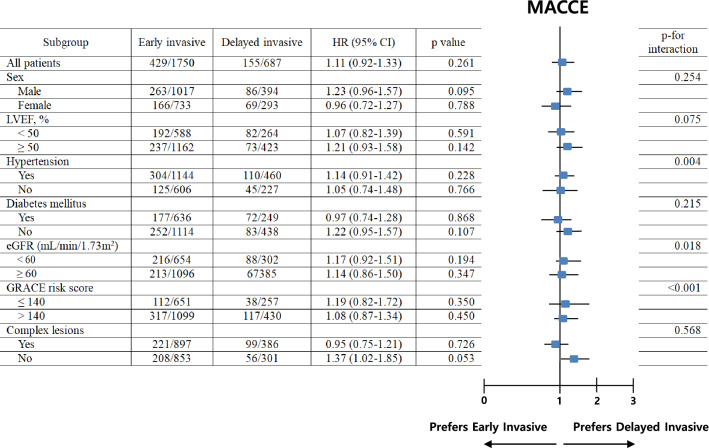
Subgroup analysis for MACCE. *MACCE* major adverse cardiac and cerebrovascular events, *HR* hazard ratio, *CI* confidence interval, *LVEF* left ventricular ejection fraction, *eGFR* estimated glomerular filtration rate, *GRACE* Global Registry of Acute Coronary Events.

The in-hospital mortality and 3-year major clinical outcomes are summarized in Table 2 and Fig. 2. In-hospital all-cause death (hazard ratio [HR] 1.581 (95% confidence interval [CI] 0.861–2.904; p = 0.140), cardiac death (CD, HR 1.924; 95% CI 0.899–4.117; p = 0.092) and non-CD (HR 1.031; 95% CI 0.368–2.892; p = 0.954) were not significantly different between the EI and DI groups. After multivariable-adjusted analysis, the 3-year major adverse cardiac and cerebrovascular events (MACCE, adjusted HR [aHR] 1.159; 95% CI 0.960–1.398; p = 0.125), all-cause death (aHR 1.180; p = 0.192), CD (aHR 1.229; p = 0.228), non-CD (aHR 1.116; p = 0.564), recurrent MI (re-MI, aHR 1.040; p = 0.881), any repeat revascularization (aHR, 1.171; p = 0.327), stroke (aHR 1.099; p = 0.713), and stent thrombosis (ST [definite or probable], aHR 2.058; 95% CI 0.690–6.143; p = 0.196) rates were not significantly different between the EI and DI groups. (Table 2). These results were confirmed after PS-matched analysis. After PS-matched analysis, the primary and secondary clinical outcomes were not significantly different between the EI and DI groups (Table 2). For further assessment of major clinical outcomes between the EI and DI groups, we compared these major clinical outcomes by limiting the study population to patients with complex lesions (Table 3). The number of patients with complex lesions in each group was more than 50% (EI vs. DI = 51.3% vs. 56.2%, p = 0.028) (Fig. 3). The MACCE rates were similar between the EI and DI groups (aHR 1.034; 95% CI 0.810–1.320; p = 0.787) (Table 3). The ST (definite or probable) rates were also similar between the EI and DI groups (aHR 2.662; 95% CI 0.531–13.35; p = 0.234). Additionally, the all-cause death, CD, non-CD, re-MI, any repeat revascularization, and stroke rates were not significantly different between the two groups after adjustment (Table 3). Figure 4 shows the subgroup analysis for MACCE. The results of subgroup analysis using Cox logistic regression model revealed that in the all subgroups except for those showing significant p-for-interaction demonstrated comparable MACCE rates in this study. Table 4 shows predictors for all-cause mortality in the total study population, which includes reduced LVEF (< 50%, aHR 1.762; 95% CI 1.414–2.195; p < 0.001), cardiogenic shock (aHR 1.984; 95% CI 1.437–2.748; p = 0.003), intra-aortic balloon pump (IABP) or extracorporeal membrane oxygenation (ECMO, aHR 3.097; 95% CI 2.010–4.771; p < 0.001), reduced renal function (aHR 2.060; 95% CI 1.625–2.612; p < 0.001), and a high GRACE risk score (> 140, aHR 2.328; 95% CI 1.716–3.159; p < 0.001).

**Table 2:** Comparison of clinical outcomes at 2 years.

Outcomes	Early invasive	Delayed invasive	Log-rank	Hazard ratio (95% CI)	p value
**All patients (Unadjusted)**	n = 1750	n = 687			
In-hospital mortality	52 (3.0)	13 (1.9)	0.136	1.581 (0.861–2.904)	0.140
Cardiac death	39 (2.2)	8 (1.2)	0.086	1.924 (0.899–4.117)	0.092
Non-cardiac death	13 (0.8)	5 (0.7)	0.954	1.031 (0.368–2.892)	0.954
3-year outcomes					
MACCE	429 (24.5)	155 (22.6)	0.261	1.111 (0.925–1.335)	0.261
All-cause death	252 (14.4)	88 (12.9)	0.295	1.138 (0.893–1.451)	0.295
Cardiac death	148 (8.5)	48 (7.1)	0.222	1.225 (0.884–1.696)	0.223
Non-cardiac death	104 (5.9)	40 (5.8)	0.854	1.035 (0.719–1.490)	0.854
Recurrent MI	66 (4.1)	26 (4.0)	0.967	1.010 (0.641–1.590)	0.967
Any repeat revascularization	153 (9.6)	52 (8.2)	0.291	1.184 (0.865–1.622)	0.292
Stroke	49 (3.0)	23 (3.6)	0.515	0.848 (0.517–1.392)	0.515
ST (definite or probable)	8 (0.5)	6 (0.9)	0.232	0.530 (0.184–1.527)	0.240
All patients (Multivariable-adjusted*)					
3-year outcomes	n = 1750	n = 687			
MACCE	429 (24.5)	155 (22.6)	0.261	1.159 (0.960–1.398)	0.125
All-cause death	252 (14.4)	88 (12.9)	0.295	1.180 (0.920–1.515)	0.192
Cardiac death	148 (8.5)	48 (7.1)	0.222	1.229 (0.879–1.719)	0.228
Non-cardiac death	104 (5.9)	40 (5.8)	0.854	1.116 (0.768–1.623)	0.564
Recurrent MI	66 (4.1)	26 (4.0)	0.967	1.040 (0.653–1.655)	0.881
Any repeat revascularization	153 (9.6)	52 (8.2)	0.291	1.171 (0.854–1.607)	0.327
Stroke	49 (3.0)	23 (3.6)	0.515	1.099 (0.665–1.815)	0.713
ST (definite or probable)	8 (0.5)	6 (0.9)	0.232	2.058 (0.690–6.143)	0.196
**Propensity score-matched patients**					
3-year outcomes	n = 657	n = 657			
MACCE	173 (26.3)	147 (22.4)	0.096	1.205 (0.967–1.501)	0.097
All-cause death	98 (15.1)	84 (12.9)	0.272	1.177 (0.880–1.576)	0.272
Cardiac death	56 (8.7)	45 (6.9)	0.256	1.255 (0.847–1.857)	0.257
Non-cardiac death	42 (6.4)	39 (6.0)	0.704	1.088 (0.704–1.682)	0.704
Recurrent MI	26 (4.3)	25 (4.1)	0.869	1.047 (0.605–1.813)	0.869
Any repeat revascularization	62 (10.3)	51 (8.4)	0.251	1.242 (0.857–1.799)	0.252
Stroke	21 (3.4)	22(3.6)	0.913	0.967 (0.532–1.759)	0.913
ST (definite or probable)	2 (0.3)	6 (1.0)	0.156	2.986 (0.603–14.80)	0.180

*MACCE* major adverse cardiac and cerebrovascular events, *CI* confidence interval, *LVEF* left ventricular ejection fraction, *BMI* body mass index, *SBP* systolic blood pressure, *DBP* diastolic blood pressure, *DM* diabetes mellitus, *PCI* percutaneous coronary intervention, *CK-MB* creatine kinase myocardial band, *eGFR* estimated glomerular filtration rate, *HDL* high-density lipoprotein, *GRACE* Global Registry of Acute Coronary Events. ^a^Adjusted by male sex, age, LVEF, BMI, SBP, DBP, cardiogenic shock, symptom-to-door time, hypertension, DM, dyslipidemia, previous MI and PCI, current smoker, peak CK-MB, peak troponin-I, serum creatinine, eGFR < 60 mL/min/1.73 m^2^, HDL-cholesterol, and GRACE risk score > 140.

**Table 3:** Comparison of clinical outcomes in patient with complex coronary lesions.

Outcomes	Early invasive (n = 897)	Delayed invasive (n = 386)	Log-rank	Unadjusted	*p*	Multivariable-adjusted^a^	*p*
				HR (95% CI)		HR (95% CI)	
MACCE	221 (24.6)	99 (25.6)	0.725	0.958 (0.756–1.215)	0.726	1.034 (0.810–1.320)	0.787
All-cause death	127 (14.2)	56 (14.5)	0.849	0.970 (0.708–1.328)	0.849	1.047 (0.755–1.452)	0.783
Cardiac death	69 (7.8)	32 (8.3)	0.707	0.923 (0.607–1.403)	0.707	1.139 (0.735–1.765)	0.561
Non-cardiac death	58 (6.4)	24 (6.2)	0.895	1.032 (0.642–1.661)	0.895	1.081 (0.661–1.768)	0.758
Recurrent MI	37 (4.4)	16 (4.5)	0.974	0.990 (0.551–1.780)	0.974	1.159 (0.634–2.119)	0.631
Any repeat revascularization	79 (9.6)	36 (10.3)	0.772	0.943 (0.636–1.399)	0.772	1.050 (0.701–1.572)	0.814
Stroke	27 (3.2)	16 (4.6)	0.294	0.719 (0.387–1.335)	0.296	1.216 (0.640–2.312)	0.550
ST (definite or probable)	4 (0.5)	3 (0.9)	0.454	0.596 (0.127–2.542)	0.460	2.662 (0.531–13.35)	0.234

*MACCE* major adverse cardiac and cerebrovascular events, *ST* stent thrombosis, *HR* hazard ratio, *CI* confidence interval, *LVEF* left ventricular ejection fraction, *BMI* body mass index, *SBP* systolic blood pressure, *DBP* diastolic blood pressure, *DM* diabetes mellitus, *PCI* percutaneous coronary intervention, *CK-MB* creatine kinase myocardial band, *eGFR* estimated glomerular filtration rate, *HDL* high-density lipoprotein, *GRACE* Global Registry of Acute Coronary Events. ^a^Adjusted by male sex, age, LVEF, BMI, SBP, DBP, cardiogenic shock, symptom-to-door time, hypertension, DM, dyslipidemia, previous MI and PCI, current smoker, peak CK-MB, peak troponin-I, serum creatinine, eGFR < 60 mL/min/1.73 m^2^, HDL-cholesterol, and GRACE risk score > 140.

**Table 4:** Predictors for all-cause mortality in the total study population.

Variables	Unadjusted		Adjusted	
	HR (95% CI)	p	HR (95% CI)	p
Early invasive vs. delayed invasive	1.138 (0.893–1.454)	0.295	1.239 (0.970–1.583)	0.086
Male	1.149 (0.924–1.428)	0.212	1.102 (0.710–1.218)	0.356
LVEF, < 50%	2.346 (1.895–2.904)	< 0.001	1.762 (1.414–2.195)	< 0.001
Cardiogenic shock	1.792 (1.209–2.655)	0.004	1.984 (1.437–2.748)	0.003
IABP or ECMO	5.578 (3.682–8.449)	< 0.001	3.097 (2.010–4.771)	< 0.001
Hypertension	1.142 (0.909–1.436)	0.254	1.022 (0.805–1.299)	0.856
Diabetes mellitus	1.549 (1.251–1.917)	< 0.001	1.189 (0.948–1.491)	0.135
Dyslipidemia	1.322 (0.898–1.946)	0.157	1.317 (0.890–1.944)	0.169
eGFR < 60 mL/min/1.73 m^2^	2.783 (2.235–3.466)	< 0.001	2.060 (1.625–2.612)	< 0.001
GRACE risk score > 140	3.413 (2.552–4.565)	< 0.001	2.328 (1.716–3.159)	< 0.001

*HR* hazard ratio, *CI* confidence interval, *LVEF* left ventricular ejection fraction, *IABP* intra-aortic balloon pump, *ECMO* extracorporeal membrane oxygenation, *eGFR* estimated glomerular filtration rate, *GRACE* Global Registry of Acute Coronary Events.

## Discussion

The main findings of this prospective, observational study were: (1) after multivariable-adjusted and PS-matched analyses, MACCE, all-cause death, CD, non-CD, re-MI, any repeat revascularization, stroke, and ST (definite or probable) rates were similar between the EI and DI groups; (2) even after limiting the study population to patients who had complex lesions, the primary and secondary clinical outcomes were not significantly different between the EI and DI groups.

Theoretically, through the EI strategy, the operator could find significant lesions earlier in patients with NSTEMI and could have the opportunity for early revascularization, salvage of ischemic myocardium, and facilitation of earlier discharge from a facility^2,10^. In contrast, DI strategy may provide adequate time for optimal medical treatment in order to decrease thrombus burden and improve plaque stability^10^. In the recent European guideline, the recommended diagnostic and interventional strategies for older patients and younger patients are the same (class I and level of evidence B)^1^. However, the optimal timing of PCI in NSTEMI remains a subject of debate. The clinical presentation of NSTE-ACS in older person is atypical^11,12^ and the electrocardiographic changes are less frequent in older than in younger patients^7,12^. Despite the significant decrease in mortality and morbidities of ACS because of evidence based therapy^13^, these improvements in ACS treatment strategy have not equally improved outcomes for older adults^2^. Regarding these characteristics^2,7,11,12^ in older people, the information dealing with the preferred treatment option between the EI and DI strategies could be important for the interventional cardiologist. In the old report, EI strategy showed significantly improved clinical outcomes compared with conservative treatment in elderly patients with NSTE-ACS^14^. However, these studies were not performed in the era of new-generation DES and that did not compare clinical outcomes between the EI and DI strategies^14,15^. Furthermore, since the available data on this subject is limited^8^, the comparative results between the EI and DI strategies in older patients with NSTEMI are limited. Hence, in this study, we investigated the long-term clinical outcomes between the EI and DI strategies in older adults with NSTEMI undergoing successful new-generation DES implantation. In our study, the major clinical outcomes were not significantly different between the EI and DI groups after adjustments (multivariable or PS-matched) during a 3-year follow-up period. An EI strategy is useful but increases the risks of stroke and bleeding, which are the main complications of this strategy^14,15^. The key study of the current guidelines^1,2^ was the TIMACS trial^3^. Since the study was performed between April 2003 and June 2008; nearly half of the cases used bare-metal stents, and the first-generation DES might be used at that time. Moreover, less than 60% of the patients underwent PCI. At 6 months, the primary outcome (a composite of death, MI, or stroke) were similar between the EI and DI groups (HR 0.85; 95% CI 0.68–1.06; p = 0.15)^3^. Although this study showed valuable results for understanding the beneficial effect of EI CAG in patients with ACS^3^, accounting for the limitations mentioned, the results of our study could be more impactful. In the most recently published registry data, the EI strategy was associated with lower all-cause death (HR 0.61; 95% CI 0.51–0.71), CD (HR 0.52; 95% CI 0.43–0.63), and MACE (HR 0.62; 95% CI 0.54–0.71) than those in the DI strategy^16^. However, similarly with TIMACS trial^3^, this study was conducted between the years 2003 and 2017. Therefore, the type of DES did not belong to the new-generation DES.

In our study, the high number of comorbidities including hypertension, diabetes mellitus, previous MI, previous heart failure, previous stroke, reduced renal function in older adults with NSTMI (Table 1) are consistent with the previously published data^8,16^. This increasing prevalence of cardiovascular disease with aging has been attributed to several age-related changes including vascular wall elasticity, coagulation and hemostatic system, and endothelial dysfunction^17–19^. Therefore, age related decline in organ function increases cardiovascular diseases^19^.

Frailty is very common in older adults with cardiovascular diseases and frailty contributes valuable prognostic insights incremental to existing risk models and assists clinicians in defining optimal care pathways for their patients^20^. In elderly NSTEMI patients, frailty was independently associated with all-cause mortality at long-term follow-up of more than 6 years^21^. In the Australian Cooperative National Registry of Acute Coronary Care, Guideline Adherence and Clinical Events (CONCORDANCE) registry^22^, increased frailty was independently associated with increased post-discharge all-cause mortality. More recent study showed that an assessment of both cognitive and physical conditions should be included in the comprehensive geriatric evaluation of hospitalized older STEMI patients^23^. Hence, Faubert et al.^24^ emphasized that the management of NSTEMI in elderly patients must be individualized with regard to the patient’s goals, comorbid conditions, overall health, and cognitive status. Mone et al.^25^ showed the importance of thrombus aspiration in the treatment of STE-myocardial infarction (STEMI) in a group of high-risk patients such as elderly with frailty.

Even though the primary and secondary clinical outcomes were not significantly different between the EI and DI groups, after adjustment, reduced LVEF, cardiogenic shock, IABP or ECMO, reduced renal function, and a high GRACE risk score were significant predictors for all-cause mortality in this study (Table 4). Hayıroğlu et al.^26^ showed the mortality rate remains high despite IABP support in patients with ACS. Çinar et al.^27^ reported that the incidence of in-hospital mortality was significantly greater in patients with a high age, creatinine, ejection fraction score compared with the intermediate or the low score group (p < 0.005) among patients with STEMI related cardiogenic shock.

To clearly estimate the long-term clinical outcomes, we performed additional analysis as shown in Table 3. Even after considering the patients with complex lesions, the 3-year major clinical outcomes were not significantly different (Table 3). Subgroup analyses for MACCE in group A and B (Fig. 4) showed that all subgroups except for those showing significant p-for-interaction had comparable MACCE rates.

We agree with the current guideline recommendations that suggest that the management of older patients should be based on ischemic and bleeding risks, estimated life expectancy, comorbidities, the need for non-cardiac surgery, quality of life, frailty, cognitive, functional impairment, patient values and preferences, and the estimated risks and benefits of revascularization^1^. Our results showed that in the era of new-generation DES, the major clinical outcomes were not significantly different between the EI and DI strategies in older adults with NSTEMI after successful stent implantation during a 3-year follow-up period. Hence, we suggested that the current guideline^1,2^ about the management of older patients with NATE-ACS with CAG and PCI needs to be reevaluated under the era of new-generation DES. In this study, although the population may have been insufficient to provide meaningful results, 20 tertiary high-volume University hospitals participated in the registry. Therefore, we believe that our results could provide helpful information to interventional cardiologists in terms of long-term effects of EI and DI strategies in older adults with NSTEMI undergoing successful implantation of new-generation DES.

This study had other limitations. First, even though this study is a prospective, observational registry, it is not a randomized controlled study; there may have been some selection bias. Moreover, the variables that were not included in the data registry might have affected the study outcome despite the multivariable and PS-matched analyses. Second, because we set the cut-off value of older adults at age ≥ 65 years in our study, our results could change according to different cut-off ages. Third, as mentioned, although bleeding is an important complication that occurs after PCI in older adults^14,15^, anti-platelet therapy after 1 year index PCI was different among the physicians; we could not include bleeding complication as an outcome parameter in our study during a 3-year follow-up period. This is a major shortcoming of our study. Fourth, the 3-year follow-up duration was insufficient to evaluate long-term adverse events. Finally, contrast induced nephropathy is an important factor and acute kidney injury can effect long-term outcomes^28^. A recent report demonstrated that acute kidney injury was an important independent prognostic factor (HR 2.244; 95% CI 1.077–4.676; p = 0.031) for 5-year mortality among patients with STEMI complicated by cardiogenic shock and treated with primary PCI^28^. However, because these variables (contrast induced nephropathy and acute kidney injury) were not included in the data registry, which could have caused significant bias.

In conclusion, in the era of new-generation DES, the major clinical outcomes were not significantly different between the EI and DI strategies in older adults with NSTEMI after successful stent implantation during a 3-year follow-up period. However, further randomized, large-scale, and long-term follow-up studies are needed to clarify the differences of the clinical outcomes between these two different reperfusion strategies in those patients.

## Methods

### Study population

A total of 13,104 patients with AMI between November 2011 and December 2015 were recruited from Korea AMI Registry-National Institute of Health (KAMIR-NIH)^29^. KAMIR-NIH is a nation-wide prospective multicenter registry integrated from 20 high-volume centers in the Republic of Korea. Detailed information on this registry can be found on the website (http://www.kamir.or.kr). All patients aged ≥ 18 years at the time of hospital admission were included. Patients who did not receive PCI (n = 1369, 10.4%) or who received unsuccessful PCI (failed PCI [n = 61, 0.5%] and suboptimal PCI [n = 94, 0.7%]), received plain old balloon angioplasty (n = 739, 5.6%), were treated with bare-metal stent or first-generation DES (n = 563, 4.3%), underwent coronary artery bypass graft (n = 38, 0.3%), had STE MI (STEMI) (n = 5342, 40.8%), and were unavailable for follow-up (n = 157, 1.2%) were excluded. Moreover, the patients aged less than 65 years (n = 2310, 48.7%) were excluded. Overall, 2437 patients with NSTEMI who underwent successful new-generation DES implantation were included (Fig. 1). The types of new-generation DES used are listed in Table 1. The definition of older adults is controversial. In general, a person is considered old if their civil age is ≥ 60 or 65 years^30^. The average age at which individuals experience a first heart attack is 65.8 years for men and 70.4 years for women^12^. Additionally, based on the Consensus Development Conference on Diabetes and Older Adults (age ≥ 65 years) convened by the American Diabetes Association in Feb 2012^31^ and other report^32^ showed that multimorbidity and polypharmacy are highly prevalent among adults aged ≥ 65 years, we set the cut-off value at ≥ 65 years for older adults in our study. These patients were divided into two groups: EI (n = 1750, 71.8%) and DI (n = 687, 28.2%) (Fig. 1). Trained research coordinators at each center collected patient data using a web-based report form on the Internet-based Clinical Research and Trial management system, supported by a grant from the Korean Centers for Disease Control and Prevention since November 2011 (URL: http://cris.nih.go.kr/cris/en/; Unique identifier: KCT0000863; First registration: 01/11/2011). The study was conducted in accordance with the ethical guidelines of the 2004 Declaration of Helsinki. The study was approved by the ethics committee of each participating center and the Chonnam National University Hospital Institutional Review Board ethics committee (CNUH-2011-172). All patients included in the study provided written informed consent prior to enrollment. They were followed-up via face-to-face interviews, phone calls, or chart reviews and they completed a 3-year follow-up schedule. All clinical events were evaluated by an independent event adjudication committee. The event adjudication process has previously been described by the KAMIR investigators^29^.

### PCI procedure and medical treatment

CAG and PCI were performed via a transfemoral or transradial approach in accordance with the general guidelines^33^. Aspirin (200–300 mg) and clopidogrel (300–600 mg), ticagrelor (180 mg), or prasugrel (60 mg) were prescribed to the patients as loading doses before PCI. After PCI, all patients were recommended to take aspirin (100 mg/day) along with clopidogrel (75 mg/day), ticagrelor (90 mg twice a day), or prasugrel (5–10 mg/day) for at least 1 year. The access site, revascularization strategy, and selection of DES were left to the discretion of the individual operators.

### Study definitions and clinical outcomes

NSTEMI was defined as the absence of persistent STE with increased levels of cardiac biomarkers and appropriate clinical context^1,2^. A successful PCI was defined as residual stenosis of < 30% and thrombolysis in MI (TIMI) flow grade 3 in the infarct-related artery. Glomerular function for estimated glomerular filtration rate (eGFR) was calculated using the Chronic Kidney Disease Epidemiology Collaboration equation^34^. The GRACE risk score^35^ was calculated for all the patients. Complex lesions were defined as PCI for unprotected left main coronary disease, multivessel PCI, multiple stents implantation (≥ 3 stents per patient), and those with the total length of deployed stent being over 38 mm^36,37^. The primary clinical outcome was the occurrence of major adverse cardiac and cerebrovascular events (MACCE), which was defined by all-cause death, recurrent MI (re-MI), any repeat coronary revascularization, including target lesion revascularization, target vessel revascularization (TVR), non-TVR, and stroke. According the American Heart Association/American Stroke Association guideline^38^, an acute cerebrovascular event resulting in death or neurological deficit for > 24 h or the presence of acute infarction demonstrated by imaging studies was defined as a stroke. An all-cause death was considered a cardiac death (CD) unless an undisputed non-cardiac cause was present^39^. The secondary clinical outcome was definite or probable stent thrombosis (ST) during a 3-year follow-up period. Stent thrombosis was defined according to the definition provided by the Academic Research Consortium^40^. The definitions of re-MI, TLR, TVR, and non-TVR have been published previously^41^.

### Statistical analysis

For continuous variables, the differences between the groups were evaluated using unpaired t-tests. Data are expressed as the mean ± standard deviation, or median (interquartile range). For discrete variables, the differences between the groups were expressed as counts and percentages and were analyzed using the chi-squared or Fisher’s exact test. Univariate analysis was performed for all variables of EI and DI groups with the *p*-value set at < 0.05. Subsequently, we performed a multicollinearity test^42^ between the included variables to confirm non-collinearity between them (Supplementary Table S1). Variance inflation factor (VIF) values were calculated to measure the degree of multicollinearity among the variables. A VIF of > 5 indicated a high correlation^43^. When the tolerance value was < 0.1^44^ or the condition index was > 10^43^, the presence of multicollinearity was considered. The variables included in the multivariable Cox regression analysis were: male sex, age, LVEF, body mass index, systolic blood pressure, diastolic blood pressure, cardiogenic shock, symptom-to-door time, hypertension, diabetes mellitus, dyslipidemia, previous MI, previous PCI, current smoker, CK-MB, peak troponin-I, serum creatinine, eGFR < 60 mL/min/1.73 m^2^, high-density lipoprotein cholesterol, and GRACE risk score > 140. Moreover, to adjust for potential confounders, propensity score (PS)-matched analysis was performed using a logistic regression model. We tested all potentially relevant variables such as baseline clinical, angiographic, and procedural factors (Table 1). The c-statistic for the PS-matched (PSM) analysis in this study was 0.724. Patients in the EI group were matched to those in the DI group (1:1) using the nearest available pair-matching method according to PSs. The subjects were matched with a caliper width of 0.01. This procedure yielded 1314 well-matched pairs (Table 1). Various clinical outcomes were estimated using a Kaplan–Meier curve analysis, and group differences were compared using the log-rank test. Statistical significance was defined as a 2-tailed p-value of < 0.05. All statistical analyses were performed using SPSS software v. 20 (IBM; Armonk, NY, USA).

## Supplementary Information

Supplementary Table S1.

## Data Availability

Data is contained with the article or supplementary material.
