# Supplementary material for: Outcomes of early versus delayed invasive strategy in older adults with non-ST-segment elevation myocardial infarction
Source: Sci Rep. 2022 Jul 6;12:11429. doi: 10.1038/s41598-022-15593-w (PMC9259558; doi:10.1038/s41598-022-15593-w)
Supplement: Supplementary file 1 — Supplementary Table S1. [file 41598_2022_15593_MOESM1_ESM.docx]

|  | Variance Inflation Factors | Tolerance | Condition Index |
| --- | --- | --- | --- |
| Male | 1.223 | 0.818 | 1.000 |
| Age | 1.392 | 0.719 | 3.370 |
| LVEF | 1.138 | 0.879 | 3.665 |
| BMI | 1.088 | 0.919 | 3.848 |
| SBP | 2.987 | 0.345 | 4.065 |
| DBP | 2.500 | 0.400 | 4.160 |
| Cardiogenic shock | 1.260 | 0.794 | 4.208 |
| Symptom-to-door time | 1.029 | 0.972 | 4.289 |
| Hypertension | 1.129 | 0.885 | 4.417 |
| Diabetes mellitus | 1.169 | 0.855 | 4.475 |
| Dyslipidemia | 1.051 | 0.952 | 4.528 |
| Previous MI | 1.649 | 0.607 | 4.676 |
| Previous PCI | 1.663 | 0.601 | 4.851 |
| Current smoker | 1.130 | 0.885 | 5.262 |
| Peak CK-MB | 1.100 | 0.909 | 5.751 |
| Peak troponin-I | 1.099 | 0.910 | 6.525 |
| Serum creatinine | 1.334 | 0.750 | 6.789 |
| eGFR <60mL/min/1.73m^2^ | 1.447 | 0.691 | 7.061 |
| HDL-cholesterol | 1.093 | 0.915 | 7.297 |
| GRACE risk score, > 140 | 1.570 | 0.637 | 7.740 |
| Clopidogrel | 11.486 | 0.087 | 8.067 |
| Ticagrelor | 9.907 | 0.101 | 8.582 |
| Prasugrel | 3.647 | 0.274 | 10.251 |
| ACEI or ARB | 1.063 | 0.940 | 16.864 |
| Multivessel disease | 1.078 | 0.927 | 19.938 |
| Pre-PCI TIMI flow grade 0/1 | 1.124 | 0.890 | 21.910 |
| Thrombus aspiration | 1.121 | 0.892 | 24.856 |
| IVUS/OCT | 1.047 | 0.955 | 31.716 |
| FFR | 1.019 | 0.981 | 32.984 |
| Stent diameter | 1.104 | 0.906 | 45.047 |
| Stent length | 2.454 | 0.407 | 48.624 |
| Number of stents | 2.405 | 0.416 | 106.818 |

**Table S1** Results of collinearity test for MACCE

*MACE* major adverse cardiac events, *LVEF* left ventricular ejection fraction, *BMI* body mass index, *SBP* systolic blood pressure, *DBP* diastolic blood pressure, *MI* myocardial infarction, *PCI percutaneous coronary intervention, CK-MB* creatine kinase myocardial band, *eGFR* estimated glomerular filtration rate, *HDL* high density lipoprotein, *GRACE* Global Registry of Acute Coronary Events*, ACEI* angiotensin converting enzyme inhibitor, *ARB* angiotensin receptor blocker, *TIMI* thrombolysis in myocardial infarction, *IVUS intravascular ultrasound, OCT* optical coherence tomography, *FFR* fractional flow reserve*.*
